# Vitamin D receptor polymorphisms and breast cancer risk in a large population-based case-control study of Caucasian and African-American women

**DOI:** 10.1186/bcr1833

**Published:** 2007-12-07

**Authors:** Britton Trabert, Kathleen E Malone, Janet R Daling, David R Doody, Leslie Bernstein, Giske Ursin, Polly A Marchbanks, Brian L Strom, Mariela C Humphrey, Elaine A Ostrander

**Affiliations:** 1 Division of Public Health Sciences, Fred Hutchinson Cancer Research Center, Fairview Ave N, Seattle, Washington 98109-1024, USA; 2 Department of Epidemiology, University of Washington School of Public Health and Community Medicine, NE Pacific St, Seattle, Washington 98195, USA; 3 Department of Preventive Medicine, Keck School of Medicine and Norris Comprehensive Cancer Center, University of Southern California, Zonal Ave, Los Angeles, California 90089, USA; 4 Department of Nutrition, University of Oslo, Sognsvannsveien, 0372 Oslo, Norway; 5 National Center for Chronic Disease Prevention and Health Promotion, Centers for Disease Control and Prevention, Clifton Rd, Atlanta, Georgia 30333, USA; 6 Center for Clinical Epidemiology and Biostatistics and Department of Biostatistics and Epidemiology, University of Pennsylvania, Guardian Drive, Philadelphia, Pennsylvania 19104, USA; 7 Divisions of Human Biology and Clinical Research, Fred Hutchinson Cancer Research Center, 1100 Fairview Ave N, Seattle, Washington 98109-1024, USA; 8 Cancer Genetics Branch, National Human Genome Research Institute, National Institutes of Health, South Drive, Bethesda, Maryland 20892, USA

## Abstract

**Introduction:**

The involvement of vitamin D receptor (VDR), which is a key mediator in the vitamin D pathway, in breast cancer etiology has long been of interest.

**Methods:**

We examined the association between polymorphisms in the 3' end of the *VDR *gene, specifically *BsmI *and *Poly(A)*, and breast cancer risk within a large, population-based, case-control study of breast cancer. Cases (*n *= 1,631) were Caucasian and African-American women, aged 35 to 64 years, who were diagnosed with incident, invasive breast cancer between July 1994 and April 1998. Control individuals (*n *= 1,435) were women without breast cancer ascertained through random digit dialing.

**Results:**

Accounting for age, study site, and sampling weights, we observed a significantly increased risk for breast cancer among Caucasian, postmenopausal carriers of the bb genotype of *BsmI *(odds ratio = 1.53, 95% confidence interval = 1.04 to 2.27). However, no associations with the bb genotype were observed in African-American women. Overall, there were no significant associations between the *Poly(A) *genotype and breast cancer risk in either racial group. Smoking status (ever/never) modified the association between both the *BsmI *and *Poly(A) *genotypes and breast cancer risk. The respective associations between these genotypes and breast cancer risk did not significantly vary by oral contraceptive use, hormone replacement therapy, or body mass index.

**Conclusion:**

Our results provide additional support for an increased risk for breast cancer in postmenopausal Caucasian women with the *BsmI *bb genotype and shed light on possible differential effects by menopausal status and race.

## Introduction

Vitamin D is a key player in cell proliferation, differentiation, and apoptosis in normal and malignant breast cells [1,2]. Indeed, the active form of vitamin D is hypothesized to have antiproliferative effects in many types of cells, including breast cancer cells [3-6]. The observation of a reduced risk for breast cancer among women with high vitamin D status lends support to this hypothesis [3,4].

Vitamin D has also been established as a determinant of bone density and, furthermore, higher bone density has been shown to be associated with risk for developing breast cancer [7-9]. Whether the association between bone density and breast cancer is directly related to vitamin D or is explained wholly or in part by other factors such as the relationship of bone density to endogenous hormones, particularly estrogen, remains unclear.

The autocrine/paracrine pathway of vitamin D biosynthesis has been implicated in breast cell carcinogenesis [1]. In this pathway, when circulating 25-hydroxyvitamin D reaches the mammary tissue it is further converted to 1,25-dihydroxyvitamin D by endogenous 1-α-hydroxylase in the breast [1,2]. The locally produced 1,25-dihydroxyvitamin D may bind to the vitamin D receptor (VDR) and regulate cell development or progression [2].

Both the 5' and 3' termini of the *VDR *gene exhibit sequence level variation in the population. Three restriction fragment length polymorphisms at the 3' end of the gene (*BsmI*, *ApaI*, and *TaqI *restriction sites) have been observed to be in linkage disequilibrium with one another and with the variable length *Poly(A) *sequence in the 3'-untranslated region [10-15]. Because of the key role that VDR plays in the vitamin D biosynthesis pathway, it has been hypothesized that polymorphisms within the *VDR *gene may modify the risk for breast cancer, either singularly or through gene-gene or gene-environment interactions.

The long sequence of *Poly(A) *has been associated with both increased and decreased risks for breast cancer [10,16,17]. Additionally, variation in length of the *Poly(A) *tract has been associated with prostate cancer risk in a subset of studies [12-14]. Studies aimed at elucidating the association between the *BsmI *polymorphism and breast cancer risk have reported positive [3,10,16], inverse [17], and null findings [18-20]. More consistent associations have been observed between the *BsmI *polymorphism and bone density [21,22].

The inconsistency in findings to date is not surprising because most studies have been limited by sample size, and there has been considerable heterogeneity in study designs. Moreover, prior studies have focused on primarily Caucasian populations; hence, little is known about the association of *VDR *polymorphisms and breast cancer risk in African-American women. The present study was undertaken to assess whether *VDR *polymorphisms *BsmI *and *Poly(A) *are associated with breast cancer risk in a large population-based case-control study of Caucasian and African-American women aged 35 to 64 years.

## Materials and methods

### Study population

This study was conducted within the National Institute of Child Health and Human Development's Women's Contraceptive and Reproductive Experiences (CARE) study [23], the details of which have been described elsewhere. In brief, five metropolitan areas in the USA, including: Atlanta, Detroit, Los Angeles, Philadelphia, and Seattle, were included in this population-based case-control study. Cases were ascertained by the Surveillance, Epidemiology, and End Results population-based cancer registries at four sites and by field staff monitoring catchment area hospital records at the fifth site. Cases consisted of Caucasian and African-American women aged 35 to 64 years, with no history of prior breast cancer, who were diagnosed with invasive breast cancer between July 1994 and April 1998. Two subgroups (younger cases [<50 years] and African-American cases) were over-sampled to achieve a generally uniform distribution across race and age strata. Centralized random digit dialing was used to ascertain population-based control individuals, consisting of women without breast cancer, and selection fractions were designed to match case interview frequencies within strata of study center, race, and 5-year age group. A total of 4,575 cases (76.5% of those eligible) and 4,682 controls (78.6% of those eligible) completed an in-person interview on breast cancer risk factors, including family history.

Of interviewed women, 33% were sampled for blood collection based on available funding. The stratified sampling plan for blood collection consisted of a blood sample from all cases and controls with a first-degree family history of breast cancer (affected mother, sister, or daughter) plus a random sample of those without a first-degree family history. Random selection for blood collection was based on sampling fractions specific to study center, race, and 5-year age group to achieve a relatively uniform distribution across strata. Of the 2,049 cases and 1,949 controls selected for blood draw, 1,644 (80.2%) cases and 1,451 (74.3%) controls donated a sample. This represents 35.9% and 31.0% of all CARE cases and controls, respectively. Among both cases and controls selected for blood collection, the proportions who gave blood did not vary by age. However, those who gave blood were proportionately more likely than those who did not give blood to be Caucasian (cases: 69.9% versus 42.0%, *P *< 0.001; controls: 68.6% versus 44.1%, *P *< 0.001), to have attended college (cases: 59.9% versus 48.0%, *P *< 0.001; controls: 57.2% versus 47.1%, *P *< 0.001), and to have local stage disease (63.5% versus 56.7%, *P *= 0.03). Additionally, among only the controls, those who gave blood were more likely than those who did not to have a positive family history of breast cancer (52.8% versus 43.6%, *P *= 0.001). The weighted sampling probabilities used in these analyses should alleviate these differences to some extent.

Of the 3,095 total blood samples, 3,066 samples (1,631 cases and 1,435 controls) provided genotyping data on one or both of the VDR polymorphisms; the other 29 samples were excluded because they did not provide data on either polymorphism. Study participants provided written informed consent for the interview and for the use of specimens for laboratory analysis.

### Genotyping

The *BsmI VDR *polymorphism is located within intron 8 of the gene. Initial PCR amplification involved 25 to 50 ng genomic DNA, purified from whole blood using standard proteinase K, phenol chloroform extraction methods [24]. The *BsmI VDR *polymorphism was determined using PCR-restriction fragment length polymorphism analysis, as we described previously [25]. PCR amplification of genomic DNA (25 ng) used 35 cycles with an annealing temperature of 66°C and the following primers: 5'-CAACCAAGACTACAAGTACCGCGTCAGTGA-3' and 5'-AACCAGCGGGAAGAGGTCAAGGG-3'.

The resulting 800 base pair (bp) PCR product is then diluted and digested with *BsmI *at 65°C for 18 hours using 5 units of enzyme (Boehringer Mannheim, Penzberg, Germany) per 20 μl reaction. Following digestion, the PCR products were separated using 2% agarose gels containing ethidium bromide and visualized under short-wave UV light. Fragments of 650 and 150 bp are visible if the 800 bp product is cut by the *BsmI *restriction enzyme.

DNA from homozygote individuals (BB) lacking a *BsmI *restriction site appeared on the gel as single 800 bp band. DNA from homozygote individuals (bb) appeared as two well-separated bands, 650 and 150 bp, indicating that the *BsmI *enzyme site is present in both alleles. Heterozygotes (Bb) have three bands: a 650 bp band and a 150 bp band (representing the presence of the *BsmI *site in one allele) plus an 800 bp band (indicating its absence in the other). Because the assay is extremely robust and it is easy to detect heterozygotes as well as both types of homozygotes, no additional quality control procedures were implemented except water blanks to check for sample contamination, standards to size restriction digestion products, and orientation markers.

The *VDR Poly(A) *polymorphism was analyzed using a nested PCR reaction [25]. In the first PCR reaction, genomic DNA (25 ng) was amplified for 35 cycles with an annealing temperature of 62°C and the following primers: 5'-GACAGAGGAGGGCGTG ACTC-3' and 5'-GTGTAGTGAAAAGGACACCGGA-3'. For the nested PCR, 5 μl from a 1:200 dilution of the first PCR reaction was amplified in the presence of 2.5 pmol IR770dATP with the following conditions: 94°C for 2 minutes, followed by 35 cycles with 30 seconds each at 94°C, 66°C, and 72°C, concluding with 3 minutes at 72°C. The primers were 5'-GAGACCAACCTGACCA-3' and 5'-CCTCAGCCTCCTGAGT-3'.

The PCR products were resolved on a Li-Cor Model 4200 automated infrared DNA sequencer (Li-Cor, Lincoln, NE, USA). Genotypes were assigned with commercially available SAGA software (Li-Cor). Allele sizes are scored by comparison with known control sizes and confirmed by rerunning with additional samples of known size.

### Statistical methods

Sampling weights were computed by dividing the total number of women interviewed in each of the 240 strata (defined by case-control status, age [5-year age categories], race [African-American, Caucasian], study site, and first-degree family history of breast cancer [present, absent]) by the number of women sampled for the genotyping. These weights allowed results from tested samples to be adjusted so that they represented the proportions and effects expected if the entire CARE study population had been tested, and were used to calculate weighted proportions and weighted odds ratios (ORs). These methods were described previously [26].

The Pearson χ^2 ^test was used to check Hardy-Weinberg equilibrium and to compare case and control distributions across covariate categories. The correlation coefficient *r *was used as the estimate of pair-wise linkage disequilibrium between *BsmI *and *Poly(A) *alleles in control women. Associations between *VDR *polymorphisms and breast cancer risk were estimated using unconditional logistic regression controlling for matching factors (age, study site, and race) and sampling weights. The *BsmI *BB and short *Poly(A) *SS genotypes were chosen as the respective referent categories based on published literature [10,16,19]. As reported previously, women were considered menopausal if they had known menopause (natural, induced, or type unclear) or assumed natural menopause [27]. Known or suspected risk factors for breast cancer (including menopausal status, first-degree family history of breast cancer, body mass index [BMI], parity, breast-feeding history, hormone therapy [HT] use [ever, former, and current use of unopposed estrogen as well as combined estrogen and progestin], oral contraceptive [OC] use [ever, former, and current use as well as duration], education, alcohol consumption, smoking, and physical activity) were evaluated for potential confounding effects. BMI was defined using World Health Organization cut points, and smoking was dichotomized as ever smokers and never smokers. The overwhelming majority (90.6%) of ever smokers reported lifetime smoking of at least one pack-year, whereas only 1.2% of ever smokers smoked 0.1 lifetime pack-years or fewer. A variable was classified as a confounding factor if the addition of the individual covariate to the baseline model resulted in more than a 10% change in the OR estimate.

We examined age, race, menopausal status and family history of breast cancer for potential modifying effects on the association between *VDR *polymorphisms and breast cancer risk. In addition, given prior reports of the possible shared etiology of breast cancer and bone density, we assessed the potential modifying effects of a subset of breast cancer risk factors, specifically OC use among premenopausal women, HT use among postmenopausal women, smoking, and BMI, that are also known or suspected of having an effect on bone mineral density. Effect modification was explored in stratified models as well as in models using multiplicative interaction terms. In the multiplicative interaction models, a variable was considered an effect modifier if the *P *value for the interaction terms was less than or equal to 0.05. All models in which effect modification was explored were initially stratified by race, a matching variable, and menopausal status because of existing effect modification.

Statistical analyses were performed using Stata/SE^® ^software (Version 9.2 for Windows; StataCorp LP, College Station, TX, USA). The study protocol was approved by institutional review boards at participating centers.

## Results

Table 1 shows the distribution of demographic and family history characteristics within the sampled study population with data for one or both of the *VDR *polymorphisms (*n *= 3,066). As expected by design, cases were similar to controls with regard to age and race, and the proportions with first-degree family history in our sampled group (37.4% cases and 23.6% controls) exceeded those in the underlying CARE study (17.0% cases and 9.7% controls). All subsequent analyses account for the sampled nature of the data, including the over-sampling of women with a first-degree family history of breast cancer, through sampling weight adjustment. For *Poly(A)*, there was excellent observer agreement in the 6% of randomly selected duplicates of genotyping results that were included for quality control purposes (κ statistic of 0.99). Similar information was not collected for *BsmI*. Laboratory personnel were blinded to case-control status.

**Table 1:** Sociodemographic characteristics, family history of breast cancer, and hormone use among cases and controls

Characteristics	Cases (*n *= 1,631)	Controls (*n *= 1,435)
Age (years)^a^		
35 to 39	306 (18.8)	230 (16.0)
40 to 44	243 (14.9)	246 (17.1)
45 to 49	260 (15.9)	236 (16.5)
50 to 54	295 (18.1)	229 (16.0)
55 to 59	269 (16.5)	265 (18.5)
60 to 64	258 (15.8)	229 (16.0)
Race^a^		
Caucasian	1143 (70.1)	987 (68.8)
African-American	488 (29.9)	448 (31.2)
Menopausal status		
Premenopausal	964 (59.1)	784 (54.6)
Postmenopausal	667 (40.9)	651 (45.4)
History of breast cancer^a^		
First-degree family history	610 (37.4)	338 (23.6)
No first-degree family history	969 (59.4)	1057 (73.7)
Adopted or unknown first degree	52 (3.2)	40 (2.8)
Body mass index (kg/m^2^)		
Underweight (<18.5)	31 (1.9)	40 (2.8)
Normal (18.5 to 24.9)	898 (55.3)	726 (50.7)
Overweight (25.0 to 29.9)	436 (26.8)	384 (26.8)
Obese (≥30)	260 (16.0)	283 (19.8)
Oral contraceptive use		
Never	358 (22.0)	274 (19.1)
Former	1190 (73.0)	1098 (76.5)
Current	82 (5.0)	63 (4.4)
Unopposed estrogen hormone therapy use^b^		
Never	1004 (61.6)	816 (56.9)
Former	130 (8.0)	129 (9.0)
Current (within 6 months)	206 (12.6)	245 (17.1)
Estrogen plus progestin hormone therapy use^b^		
Never	1004 (61.6)	816 (56.9)
Former	75 (4.6)	91 (6.3)
Current (within 6 months)	241 (14.8)	187 (13)
Smoking status		
Ever	893 (54.8)	769 (53.6)
Never	738 (45.2)	666 (46.4)

Proportions in this table reflect the over-sampling of younger cases, African American cases, and women with a family history of breast cancer. Values are expressed as *n *(%). ^a^Cases and controls were sampled according to race, age, study site, and family history of breast cancer. ^b^Percentages do not sum to 100% because of missing values.

We did not observe deviation from the expected Hardy-Weinberg frequencies for the *Poly(A) *genotypes in the controls (*P *= 0.69, *P *= 0.47, and *P *= 0.48 for combined controls, Caucasian controls, and African-American controls, respectively). However, we did observe significant deviation for the *BsmI *genotype (*P *< 0.01, *P *< 0.01, and *P *= 0.03 for combined controls, Caucasian controls, and African-American controls, respectively). *BsmI *and *Poly(A) *genotypes were in linkage disequilibrium for the Caucasian but not the African-American population (Caucasian: *r*^2 ^= 0.84; African American: *r*^2 ^= 0.47).

### *BsmI *and *Poly(A) *genotypes and breast cancer risk

We found relatively similar *VDR *polymorphism genotype frequencies in cases and controls (Table 2). Allelic frequencies in Caucasians were similar to those reported in other Caucasian populations (data not shown) [10,16,18]. We did not observe any associations between polymorphisms in *BsmI *or *Poly(A) *and breast cancer risk for the combined study population (overall and within racial groups) after controlling for sampling weights and matching factors: age, race, and study site. Similarly, we did not observe any associations between polymorphisms in *BsmI *or *Poly(A) *and breast cancer risk within strata of tumor stage, grade, histology, or receptor status (data not shown). After controlling for many known and suspected risk factors for breast cancer, we found no evidence that any of the variables mentioned above acted as confounding factors of these results. However, given the established variation in breast cancer risk factor profiles by menopausal status and because of the difference in the direction of the ORs by race, all further results are stratified by menopausal status and race (Table 3).

**Table 2:** Genotype and allele proportions for breast cancer risk associated with *BsmI *and *Poly(A) *genotypes

Genotype		Controls^a ^(n [%])	Allele frequency	Cases^a ^(n [%])	Allele frequency	OR^b ^(95% CI)
*BsmI*						
All	B/B	308 (21.7)		345 (20.6)		1.0 (referent)
	B/b	539 (37.2)	B 0.41	606 (37.2)	B 0.40	1.1 (0.9 to 1.3)
	b/b	564 (41.0)	b 0.59	670 (42.2)	b 0.60	1.1 (0.9 to 1.4)
	any b	1,103 (78.3)		1,276 (79.4)		1.2 (0.9 to 1.3)
Caucasian	B/B	258 (27.3)		278 (24.5)		1.0 (referent)
	B/b	371 (38.2)	B 0.46	432 (37.9)	B 0.43	1.1 (0.9 to 1.4)
	b/b	336 (34.5)	b 0.54	426 (37.5)	b 0.57	1.2 (0.9 to 1.6)
	any b	707 (84.4)		858 (84.8)		1.2 (0.9 to 1.4)
African American	B/B	50 (11.7)		67 (13.3)		1.0 (referent)
	B/b	168 (35.5)	B 0.30	174 (36.0)	B 0.32	0.9 (0.6 to 1.4)
	b/b	228 (52.8)	b 0.70	244 (50.7)	b 0.68	0.9 (0.5 to 1.3)
	any b	390 (88.4)		406 (86.7)		0.9 (0.6 to 1.3)
*Poly(A)*						
All	S/S	176 (12.6)		202 (13.1)		1.0 (referent)
	L/S	651 (45.9)	S 0.36	713 (44.2)	S 0.35	0.9 (0.7 to 1.2)
	L/L	575 (41.4)	L 0.64	665 (42.7)	L 0.65	1.0 (0.8 to 1.3)
	any L	1,226 (87.4)		1,378 (86.9)		1.0 (0.8 to 1.2)
Caucasian	S/S	149 (15.6)		167 (15.2)		1.0 (referent)
	L/S	402 (49.5)	S 0.39	532 (46.1)	S 0.38	1.0 (0.7 to 1.3)
	L/L	354 (34.9)	L 0.61	440 (38.7)	L 0.62	1.1 (0.8 to 1.5)
	any L	836 (72.7)		972 (75.5)		1.0 (0. 8 to 1.4)
African American	S/S	27 (6.8)		35 (8.9)		1.0 (referent)
	L/S	169 (39.0)	S 0.27	181 (40.5)	S 0.28	0.8 (0.5 to 1.5)
	L/L	221 (54.2)	L 0.73	225 (50.6)	L 0.72	0.7 (0.4 to 1.3)
	any L	396 (93.2)		418 (91.1)		0.8 (0.4 to 1.4)

^a^Proportions are weighted for age, race, study site, and first-degree family history sampling probabilities. ^b^Odds ratio (OR) adjusted for age, race, study site, and sampling weights. CI, confidence interval.

**Table 3:** Odds ratios and 95% confidence intervals for breast cancer risk associated with *BsmI *and *Poly(A) *genotypes, stratified by race and menopausal status

Genotype		Menopausal status	Caucasian			African-American		
			Case (%)	Control (%)	OR^a ^(95% CI)	Case (%)	Control (%)	OR^a ^(95% CI)
*BsmI*	B/B	Premenopausal	164 (25.3)	133 (25.8)	1.0 (referent)	38 (12.1)	22 (9.6)	1.0 (referent)
	B/b		244 (36.9)	198 (36.0)	1.1 (0.8 to 1.5)	108 (34.6)	90 (36.7)	0.8 (0.4 to 1.4)
	b/b		247 (37.8)	199 (38.1)	1.0 (0.7 to 1.4)	156 (53.3)	126 (53.7)	0.8 (0.4 to 1.5)
	Any b		491 (74.7)	397 (74.2)	1.0 (0.8 to 1.4)	264 (87.9)	216 (90.4)	0.8 (0.4 to 1.4)
	B/B	Postmenopausal	114 (23.5)	125 (29.2)	1.0 (referent)	29 (15.2)	28 (14.3)	1.0 (referent)
	B/b		188 (39.3)	173 (40.9)	1.2 (0.8 to 1.8)	66 (38.0)	78 (34.0)	1.0 (0.5 to 2.0)
	b/b		179 (37.2)	137 (30.0)	1.5 (1.0 to 2.3)^b^	88 (46.8)	102 (51.7)	0.9 (0.5 to 2.0)
	Any b		367 (76.5)	310 (70.8)	1.4 (1.0 to 1.9)	154 (84.8)	180 (85.7)	1.0 (0.5 to 1.8)
*Poly(A)*	S/S	Premenopausal	98 (15.7)	79 (15.4)	1.0 (referent)	23 (9.1)	14 (6.8)	1.0 (referent)
	L/S		303 (45.3)	260 (47.3)	0.9 (0.6 to 1.4)	109 (39.6)	88 (39.2)	0.8 (0.4 to 1.7)
	L/L		256 (39)	203 (37.3)	1.0 (0.7 to 1.5)	144 (51.3)	118 (54.1)	0.7 (0.3 to 1.6)
	Any L		559 (84.3)	463 (84.6)	1.0 (0.7 to 1.4)	253 (90.9)	206 (93.3)	0.7 (0.4 to 1.6)
	S/S	Postmenopausal	69 (14.5)	70 (15.8)	1.0 (referent)	12 (8.5)	13 (6.8)	1.0 (referent)
	L/S		229 (47.2)	222 (52.2)	1.0 (0.7 to 1.6)	72 (41.9)	81 (38.9)	0.8 (0.3 to 2.1)
	L/L		184 (38.3)	151 (31.9)	1.3 (0.9 to 2.1)	81 (49.5)	103 (54.3)	0.8 (0.3 to 2.1)
	Any L		413 (85.5)	373 (84.2)	1.2 (0.8 to 1.8)	153 (91.5)	184 (93.2)	0.8 (0.3 to 2.0)

^a^Odds ratio (OR) adjusted for age, race, study site, and sampling weights. ^b^*P *for trend <0.05.

The *BsmI *genotype was not associated with breast cancer risk in premenopausal Caucasian women or in African-American women. Among postmenopausal Caucasian women, the risk for breast cancer was increased in women carrying the homozygous bb genotype as compared with carriers of the BB genotype (OR = 1.5, 95% confidence interval [CI] = 1.0 to 2.3) and, although not statistically significant, the risk in women carrying the heterozygous Bb genotype was also increased (OR = 1.2, 95% CI = 0.8 to 1.8). A trend of increasing risk with increasing number of b alleles carried was statistically significant (*P *for trend = 0.03). We did not observe a significant association with breast cancer in any racial-menopausal status subgroup in relation to the *Poly(A) *polymorphism.

Because the *Poly(A) *and *BsmI *genotypes among African-American women were not in linkage disequilibrium, we explored the combined effects of the *Poly(A) *and *BsmI *genotypes in this subpopulation (results not shown). An increased or decreased risk for breast cancer was not associated with the combination of the *Poly(A) *and *BsmI *genotypes. Stratifying or adjusting for age, menopausal status, or family history of breast cancer did not alter significantly the observed absence of association.

### *BsmI *and *Poly(A) *genotypes, effect modification, and breast cancer risk

Effect modification of the relationship of either genotype with risk for breast cancer by *a priori *hypothesized potential effect modifiers (namely OC use, HT use, BMI, family history, and age), as well as stratification by tumor characteristic (grade, histology, stage, estrogen/progesterone receptor status) was not meaningful. Smoking was the only *a priori *hypothesized potential effect modifier that significantly modified the association between polymorphisms in *BsmI *or *Poly(A) *and breast cancer risk.

Smoking status significantly modified the association between the *BsmI *genotype and breast cancer risk among postmenopausal Caucasian women (*P *= 0.04). Among postmenopausal Caucasian women who reported never smoking, the risk for breast cancer in carriers of one or two b alleles was 2.2 times greater than for BB carriers (OR = 2.2, 95% CI = 1.3 to 3.9), whereas among ever smokers (current or former) the risk for breast cancer was unrelated to *BsmI *genotype. Smoking status did not significantly modify the association between *BsmI *genotype and breast cancer risk among premenopausal Caucasian women (*P *= 0.21).

Among premenopausal African-American women the *P *value for interaction between genotype and smoking status was significant (*P *= 0.04) whereas the *P *value for the interaction approached but did not achieve significance for postmenopausal African-American women (*P *= 0.08). Among postmenopausal African-American women who reported never smoking, the risk for breast cancer for carriers of one or two b alleles compared with individuals carrying the *BsmI *BB genotype was increased, albeit not statistically significantly (OR = 1.8, 95% CI = 0.7 to 4.9), whereas among premenopausal African-American women who reported never smoking the risk for breast cancer in carriers of one or two b alleles was significantly decreased compared with BB carriers (OR = 0.4, 95% CI = 0.2 to 0.9).

Smoking status also significantly modified the association between *Poly(A) *genotype and breast cancer risk among postmenopausal Caucasian women (*P *= 0.02). Among postmenopausal Caucasian women who reported never smoking, the risk for breast cancer for carriers of any L allele was 2.3 times greater than that for carriers of the *Poly(A) *SS genotype (OR = 2.3, 95% CI = 1.2 to 4.3), whereas among never smokers the risk for breast cancer was unrelated to *Poly(A) *genotype (OR = 0.7, 95% CI = 0.4 to 1.2).

In terms of the main effect of smoking, premenopausal Caucasian women who reported ever smoking had a statistically significant increased risk for breast cancer (OR = 1.4, 95% CI = 1.1 to 1.8) as compared with women who reported never smoking. Smoking was not associated with an increased risk for breast cancer among postmenopausal Caucasian women, premenopausal African-American women, or postmenopausal African-American women.

## Discussion

Within the CARE study population, we observed a slight increase in risk for breast cancer among postmenopausal Caucasian women with the *BsmI *bb genotype. There was little evidence of a similar relationship in premenopausal or African-American women. In addition, there was no evidence of a relationship between the *Poly(A) *LL genotype and risk for breast cancer overall, or within any racial or menopausal status subgroup. We do note that genotype frequencies were different by menopausal status; however, there is no biologic explanation for this difference. Automated genotyping systems, to the degree that they are flawed, have a tendency to overcall homozygotes and undercall heterozygotes, but there is no reason to assume that bb or BB genotypes would be differentially miscalled according to any exposure, including menopausal status. This observation does not change the overall conclusions of the study, and the differing results for premenopausal and postmenopausal Caucasian women are probably accounted for by small numbers.

Prior studies of *VDR *and breast cancer risk have been limited in number and yielded inconsistent results. Three hospital-based case-control studies of Caucasian women in the UK reported an increased risk for breast cancer in carriers of *BsmI *bb genotype [3,10,16]. However, these reports appear likely to involve overlapping populations. All three studies involved the same core group of authors and included patients from St. George's Hospital Medical School in London. Two used controls from the UK National Breast Screening Programme [10,16], whereas the third used controls from a screening clinic [3]. The largest of these studies [16] included 398 incident and prevalent breast cancer cases and 427 screened, disease-free controls. The investigators reported a 1.9-fold increased risk for breast cancer among carriers of the *BsmI *bb genotype, and a nearly twofold increase in risk for carriers of the *Poly(A) *LL genotype, when controlling for age, HT, and menopausal status. Participants were matched for age at sampling (controls) versus diagnosis (cases), but they differed significantly in terms of menopausal status and HT use. The second study [10] included 181 breast cancer cases and 241 controls from the same two institutions described above. Cases were not age-matched to controls. Controls were confirmed as having no detectable cancer at the time of sampling, although 89 had 'other' breast conditions such as calcifications, fibrocystic disease, or benign lumps. The authors reported a more than twofold increase in risk for breast cancer associated with both the *BsmI *bb genotype and *Poly(A) *LL genotype. The differences between this study and our own population-based study are significant, limiting our ability to compare results. The third study [3] was distinguished by the fact that the investigators also measured serum 25-hydroxyvitamin D levels. In this hospital-based, case-control study of 179 cases matched to 179 disease-free control volunteers by age, menopausal status (where possible), and time of year, a significant doubling in the risk for breast cancer was observed in women with *BsmI *bb compared with those with the BB genotype. The authors concluded that low circulating 25-hydroxyvitamin D levels, either alone or in combination with the *BsmI *Bb or bb genotype, may increase breast cancer risk.

In contrast, several prior studies did not report an increased risk for breast cancer associated with either the *BsmI *bb genotype or the *Poly(A) *LL genotype [17-20,28]. One of the largest, that conducted by Chen and colleagues [18], was conducted to investigate the role played by *VDR *polymorphisms and breast cancer risk in a nested case-control study of largely Caucasian women aged 43 to 69 years within the Nurses' Health study. The authors genotyped 1,180 cases and 1,547 controls for the *BsmI *and 1,234 cases and 1,676 controls for the *Fok1 *polymorphisms. They observed a significantly increased risk for breast cancer among carriers of the ff genotype of *Fok1 *(OR = 1.34, 95% CI = 1.06 to 1.69) as compared with those with the FF genotype, but they found no association between the *BsmI *polymorphism and breast cancer risk (OR = 0.93; 95% CI = 0.72 to 1.20) for BB versus bb. The conclusions did not change when the data were stratified by menopausal status. The *Poly(A) *variant was not tested.

To our knowledge, no prior studies of the *VDR *polymorphism and breast cancer risk have included a substantial number of African-American women. It is important to note that we did not observe a statistically significant increased risk for breast cancer associated with either the *BsmI *bb genotype or the *Poly(A) *LL genotype among the African-American women in our study. The (nonsignificant) reduced risk for breast cancer associated with carrying either the *BsmI *Bb or bb genotypes among never smokers, observed in African-American women, was probably due to small numbers in the referent category and not the presence of the allele or the effect of not smoking. Significantly larger studies of African-American women would be needed to address that question more completely.

Genetic factors such as the *VDR *polymorphism that influence disease risk may be subject to environmental influences. For example, smoking is an inconsistent risk factor for breast cancer [29], but the effects of polymorphisms in other genes on breast cancer risk have been observed to vary by smoking history [30,31]. Although not consistent in all subgroups, we did observe that the presence of any b allele elevated the risk for breast cancer among never smokers and that the relationship between the *VDR *polymorphisms and breast cancer risk varied among postmenopausal Caucasian women who reported ever or never smoking. The effect modification results should, however, be interpreted with caution; although we did observe an association between breast cancer and the *BsmI *bb genotype among postmenopausal Caucasian women, we did not observe an association between breast cancer and smoking in the same subgroup.

Vitamin D itself is proposed to have anticancer properties [32-34]. Indeed, it has been shown that calcitriol (1,25-dihydroxyvitamin D_3_), a hormonal derivative of vitamin D_3_, has an antiproliferative and prodifferentiation effect on several cell types, specifically the squamous cells of the head and neck [35]. In a study of prostate cancer, Ma and coworkers [13] reported that vitamin D intake modified the risk for prostate cancer that was associated with *VDR *polymorphism (*BsmI*). Specifically, they observed a 57% reduction in risk among men with a plasma 25-hydroxyvitamin D level below the median who had the BB versus bb genotype (relative risk = 0.43, 95% CI = 0.19 to 0.98). The risk reduction was strongest among older men (relative risk = 0.18, 95% CI = 0.05 to 0.68).

We did not have information on vitamin D levels, via either dietary intake or metabolism from routine sun exposure. However, several studies [7-9] support a relationship between an increase in breast cancer risk and bone density. Two prospective cohort studies have reported an association between greater bone mass and breast cancer risk [7,8], and one of these studies suggests that vitamin D intake can modify the risk of osteoporosis in women being treated for breast cancer [7]. In addition, men undergoing androgen ablation therapy experience osteoporosis [36,37], which also supports a relationship between sex hormones and bone modeling. It is possible that the increased bone density observed in women with an increased risk of breast cancer may simply be a marker for high levels of vitamin D uptake, reflecting specific vitamin D receptor genotypes, or perhaps a marker of higher levels of endogenous estrogens.

Our data support a role for the *BsmI *b allele in the development of breast cancer in women who never smoked, but not in those who did smoke. Why a subset of *VDR *genotypes would have a differential effect on smokers versus nonsmokers is unclear. One hypothesis is that there is some as yet undefined interaction between estrogen metabolism and smoking that is exacerbated by specific levels of vitamin D status.

The strengths of the present study include its large size, generalizability, and the ability to control for most of the established risk factors for breast cancer. To date, this is the largest study of *VDR *polymorphisms and breast cancer risk. The population-based design, wider age range, and inclusion of both African-American and Caucasian women allow a more comprehensive portrayal of the frequency of mutations in the general population than has been available to date. Despite the generous sample size, the number of women with referent genotypes was small, resulting in an inability to make precise estimates. In addition, the study was limited by the fact that the gene was interrogated at only two positions at the 3' end of the gene. Newer technologies now permit a more comprehensive approach to genotyping, and variation within the entire gene will be readily examined in subsequent studies. The departure from Hardy-Weinberg equilibrium that we observed for the *BsmI *polymorphism could be attributed to any number of factors, including undetected ethnic diversity, biologic explanations, or genotyping misclassifications. We did not include blinded duplicates in the genotyping dataset, and therefore we did not have the opportunity to check for systematic genotyping errors.

One difficulty in studies of single nucleotide polymorphisms, such as this, is the issue of whether the variant being tested is truly associated with the disease or whether there is another polymorphism in linkage disequilibrium with the polymorphism under consideration that could be responsible for the observed association. Only when the whole gene can be examined along with associated regulatory elements can we develop a clearer picture of the underlying biology that consistently links the *VDR *gene to cancer risk.

## Conclusion

This study provides additional support for an increased risk for breast cancer associated with the *BsmI *polymorphism at the 3' end of the *VDR *gene in postmenopausal Caucasian women and sheds light on the differential risk observed in menopausal status and race. Contributions of vitamin D intake to risk for breast cancer and bone mineral density, as well as potential effect modification by history of smoking, should be further studied to elucidate the precise biologic mechanisms.

## Abbreviations

BMI = body mass index; bp = base pairs; CARE = Contraceptive and Reproductive Experiences; HT = hormone therapy; OC = oral contraceptive; OR = odds ratio; PCR = polymerase chain reaction.

## Competing interests

The authors declare that they have no competing interests.

## Authors' contributions

BT performed the statistical analysis and drafted the manuscript. KM participated in study design and coordination, hypothesis generation, and manuscript preparation and also oversaw data collection and statistical analyses. JD, LB, PM, and BS participated in study design, data collection, and manuscript review. DD participated in the design of the study, manuscript review, and performed statistical analyses related to sampling. GU participated in manuscript review. MH carried out molecular genetic analyses. EO oversaw molecular genetic analyses, participated in study design, hypothesis generation, and manuscript preparation. All authors read and approved the final manuscript.
